# Disrupted structural connectivity and less efficient network system in patients with the treatment-naive adult attention-deficit/hyperactivity disorder

**DOI:** 10.3389/fpsyt.2023.1093522

**Published:** 2023-03-16

**Authors:** Takashi Ohnishi, Wataru Toda, Shuntaro Itagaki, Aya Sato, Junya Matsumoto, Hiroshi Ito, Shiro Ishii, Itaru Miura, Hirooki Yabe

**Affiliations:** ^1^Medical Affairs Division, Janssen Pharmaceutical K.K., Tokyo, Japan; ^2^Department of Neuropsychiatry, Fukushima Medical University, Fukushima, Japan; ^3^Department of Pathology of Mental Diseases, National Institute of Mental Health, National Center of Neurology and Psychiatry, Tokyo, Japan; ^4^Department of Radiology and Nuclear Medicine, Fukushima Medical University, Fukushima, Japan

**Keywords:** adult ADHD, diffusion tensor imaging, connectome, connectivity, graph theory

## Abstract

**Introduction:**

Attention-deficit/hyperactivity disorder (ADHD) is a neuropsychiatric disorder whose primary symptoms are hyperactivity, impulsivity, and inattention. Historically, ADHD was recognized as a disease of childhood and adolescence. However, many patients are known to have persistent symptoms into adulthood. Many researchers consider the neuropathology of ADHD to be based on abnormalities in multiple parallel and intersecting pathways rather than a single anatomical area, but such alterations remain to be clarified.

**Methods:**

Using diffusion tensor imaging, we investigated differences in the global network metrics estimated by graph theory and the degree of connectivity between adjacent voxels within a white matter (WM) fascicle defined by the density of the diffusing spins (connectometry) between 19 drug-naive Japanese patients with adult ADHD and 19 matched healthy controls (HCs). In adult patients with ADHD, we examined the relationships between the symptomatology of ADHD and global network metrics and WM abnormalities.

**Results:**

Compared with HCs, adult patients with ADHD showed a reduced rich-club coefficient and decreased connectivity in widely distributed WMs such as the corpus callosum, the forceps, and the cingulum bundle. Correlational analyses demonstrated that the general severity of ADHD symptoms was associated with several global network metrics, such as lower global efficiency, clustering coefficient, small worldness, and longer characteristic path length. The connectometry revealed that the severity of hyperactive/impulsive symptoms was associated with overconnectivity in the corticostriatal, corticospinal, and corticopontine tracts, the inferior fronto-occipital fasciculus, and the extreme capsule but dysconnectivity in the cerebellum. The severity of inattentive symptoms was associated with dysconnectivity in the intracerebellar circuit and some other fibers.

**Conclusion:**

The results of the present study indicated that patients with treatment-naive adult ADHD showed disrupted structural connectivity, which contributes to less efficient information transfer in the ADHD brain and pathophysiology of ADHD.

**Trial registration:**

UMIN Clinical Trials Registry (UMIN-CTR) UMIN000025183, Registered: 5 January 2017.

## 1. Introduction

Attention-deficit/hyperactivity disorder (ADHD) is a neuropsychiatric disease that usually appears in childhood and is characterized by hyperactivity, increased impulsivity, and developmentally inappropriate inattention. ADHD is known to affect children; however, a recent World Health Organization (WHO) survey on global mental health found that the prevalence of patients with adult ADHD was 2.8% on average (1). Growing evidence suggests that structural abnormalities in the brain may contribute to the pathophysiology of ADHD (2). However, a recent meta-analysis of functional and structural MRI studies on children and adolescents with ADHD found no significant convergence across structural and functional regional alterations in ADHD, suggesting that the pathophysiology of ADHD might be based on network interactions rather than a regional abnormality (3). Indeed, a recent study demonstrated that the neuropathology of ADHD is based on multiple parallel and intersecting pathways rather than a single anatomical area, which demonstrated altered functional connectivity in ADHD brains (2).

Diffusion tensor imaging, which is believed to be an indicator of fiber tract integrity, reflecting coherence, organization, and/or density of fiber bundles in the white matter of the brain, is a promising *in vivo* method that has made it possible to investigate white matter abnormalities in neuropsychiatric disorders (4). According to a recent meta-analysis, the most common WM abnormality in ADHD was a decrease in fractional anisotropy (FA) in the corpus callosum splenium (5). However, DTI studies of patients with adult ADHD have been limited, and the results are relatively inconsistent (6, 7). A possible factor contributing to inconsistent findings in adult patients with ADHD is differences between the ADHD participants with a history of stimulant treatment, such as the difference observed on the starting period of the administration of methylphenidate (MPH) (6). A previous randomized study investigating the effects of MPH treatment in male patients with ADHD reported that MPH affects FA in association tracts of the left hemisphere and the corpus callosum in children but not in adolescent and adult patients (8). Similar to the human study, an experimental study in adolescent rats reported increases in FA values in the corpus callosum after MPH administration (9). Furthermore, a study showed that the striatal genes associated with axonal myelination are upregulated by the administration of MPH in juveniles (10). Since patients with adult ADHD often consume stimulants for prolonged periods (6), such data indicated that WM integrity is affected by the treatment of MPH, and studies involving medication-naive patients with adult ADHD should be undertaken to understand the pathophysiology of adult ADHD.

However, DTI studies conducted on medication-naive patients with adult ADHD are still limited (6). Moreover, a previous DTI study estimated FA with the region of interest method and tract-based spatial statistics (6). Therefore, to conduct more comprehensive analyses to investigate WM abnormalities and its associated abnormal brain network topology in treatment-naive patients with adult ADHD, we applied conventional graph theory analysis for connectomics and the “local connectometry” (11) to investigate differences in global network metrics estimated by graph theory and the degree of connectivity between adjacent voxels within a WM fascicle defined by the density of the diffusing spins (11) between healthy controls (HCs) and patients with adult ADHD. Furthermore, we examined relationships between the symptomatology of ADHD and global network metrics and WM abnormalities. Diffusion MRI connectometry was used to derive the correlational tractography that has qualitative anisotropy (QA) correlated with the diagnosis effect and symptom severity of ADHD (11). Diffusion MRI connectometry is a method to estimate the diffusion quantities along the local tract orientation (11).

We hypothesize that, similar to untreated children with ADHD, untreated patients with adult ADHD show disintegrity of the white matter which is involved in the impairment of complex networks in the brain and contributes to the pathogenesis of adult patients with ADHD.

## 2. Methods

### 2.1. Participants

The call for participants was opened on 1 February 2017 and closed with the entry of the final participant on 1 June 2022. Patients with adult ADHD were recruited not only from the outpatient unit of psychiatry at the Fukushima Medical University but also from neighboring psychiatric hospitals; after explaining the purpose of the study, potential study participants were referred to the outpatient unit of psychiatry at Fukushima Medical University. In the current study, 20 patients with treatment-naive adult ADHD (M:F = 11:9) and 20 healthy controls (HCs) (M:F = 11:9) participated. From the Fukushima Medical University Hospital's outpatient service, we recruited participants with adult ADHD. At least two qualified psychiatrists diagnosed patients using the criteria outlined in the Diagnostic and Statistical Manual of Mental Disorders, Fifth Edition (DSM-5). Participants with comorbid psychiatric disorders other than ADHD were excluded using the structured clinical interview for the DSM-5 clinical version. The healthy controls were recruited through local advertisements at Fukushima Medical University. Participants having neurological or medical conditions that could potentially affect the central nervous system, such as atypical headache, history of head trauma with loss of consciousness, thyroid disease, epilepsy, seizures, substance-related disorders, or mental retardation, were excluded. Participants with a history of illicit drug use, antidepressants (at least not over the last 3 months), or other psychoactive medication were excluded. The level of intellectual performance of the participants was evaluated with the Japanese version of the Wechsler Adult Intelligence Scale III (WAIS-III) test. The WAIS-III test provides a standardized full-scale intelligence quotient (IQ) based on subtests that measure the level of verbal [verbal IQ (VIQ)] and non-verbal knowledge and reasoning [performance IQ (PIQ)]. We obtained written informed consent from all participants before participation. The presence and severity of ADHD symptoms in both ADHD and HCs were assessed using the Conners' Adult Attention-Deficit/Hyperactivity Disorder Rating Scale (CAARS). The Research Ethics Committee of Fukushima Medical University approved this study (Approval code: no. 2693, Approval data: 13 April 2016). The study was organized and carried out in line with the Declaration of Helsinki.

Due to the poor image quality of the diffusion tensor imaging, two participants (one ADHD and one HC) were excluded from the analyses.

### 2.2. MRI acquisition and preprocessing for connectometry analysis and graph theoretical analysis

The MRI data were acquired on a Siemens 3T Biograph mMR scanner with a 12-channel phased array coil. DTI acquisition involved a single-shot, spin-echo planar imaging sequence in contiguous axial planes that covered the whole brain. Diffusion-sensitizing gradients were applied in 30 non-collinear directions, together with acquisition without diffusion weighting (*b* = 0). The imaging parameters were set to the following values: TR = 10,100 ms, TE = 78 ms, average = 1, b-value = 1,000 s/mm^2^, slice thickness = 2.5 mm, and 60 slices. The matrix resolution was acquired at 96 × 96 and reconstructed to 96 × 96. The resolution was 2.5 × 2.5 × 2.5 mm^3^.

DICOM files were converted to SRC files by using DSI Studio. The SRC files were examined during the quality control procedure (12), and the data from two participants were excluded because they did not pass the quality control check. The SRC files were reconstructed in the MNI space using q-space diffeomorphic reconstruction (13) to obtain the spin distribution function (14). Since the b-value of acquired DTI was lower than 4,000 s/mm^2^, we applied the advanced option of “no high b for DTI.” A diffusion sampling length ratio of 1.25 was used. The output resolution in diffeomorphic reconstruction was 2.5-mm isotropic. The restricted diffusion was quantified using restricted diffusion imaging (15). The tensor metrics were calculated using DWI (11) with a b-value of lower than 1,000 s/mm^2^. The quantitative anisotropy (QA) was extracted as the local connectome fingerprint (16) and used in the connectometry analysis.

### 2.3. Data analyses

As the sample size of this study was relatively small, the normal distribution and equal variances were evaluated using the Shapiro–Wilk test and Levene's test, and if these were not guaranteed, then non-parametric tests such as the Mann–Whitney *U*-test and Spearman's rank-order correlation were used.

#### 2.3.1. Statistical analysis for demographic data

The chi-squared tests were used for gender and handedness, and independent two-sample *t*-tests were used for age, IQ, and CARRS between patients with adult ADHD and HCs. Statistical analyses were performed using R Statistical Software Version 3.1.0 (Foundation for Statistical Computing, Vienna, Austria).

#### 2.3.2. Network construction and analysis

The connectivity matrix (adjacency matrix) and graph theoretical analyses were conducted using DSI Studio and the brain connectivity toolbox (12, 17). The following procedures were used in the graph theoretical analysis. The first step was to create a tractography map from the DTI data, which included reading and parsing digital imaging and communications in medicine files, reconstructing it to characterize the main diffusion directions of the fibers and fiber tracking (11). The next step was to generate a connectivity matrix, which was calculated using the counts of the connecting tracts (11). The Desikan–Killiany–Tourville atlas was used for brain parcellation. This step included obtaining the whole-brain fiber tracks, placing seeding regions in the whole brain and spatial normalization, defining the region of interest, and creating a connectivity matrix (11). The graph theoretical network measures were then calculated from the connectivity matrix by using the brain connectivity toolbox (brain-connectivity-toolbox.net) (17). A graph is defined as a set of nodes and the edges or lines between them. The threshold was 0.001 to filter out matrix entries with a small number of connecting tracks. It is a ratio to the maximum connecting tracks in the connectivity matrix. The topology of graphs can be quantitatively described by a variety of measures. In this study, we used weighted graphs and evaluated the following global network metrics (17): (1) graph density, (2) global efficiency, (3) clustering coefficient, (4) characteristic path length, (5) small worldness, and (6) rich-club coefficient (18).

An independent two-sample *t*-test or a Mann–Whitney *U*-test was used to evaluate the diagnostic effect on each global network metric. Pearson's correlation coefficient or Spearman's rank-order correlation was computed to assess the relationship between scores of ADHD symptoms (T-score of CAARS, DSM-IV inattentive symptoms, DSM-IV hyperactive/impulsive, DSM-IV total symptoms, and ADHD index), and global network metrics. These statistical analyses were performed using R Statistical Software Version 3.1.0 (Foundation for Statistical Computing, Vienna, Austria).

#### 2.3.3. Local connectometry

Local connectometry was conducted using DSI Studio (11). To test the diagnostic effect, a non-parametric Spearman partial correlation was used to derive the correlation, and the effect of FIQ was removed using a multiple regression model. To explore the relationship between symptomatic severity in adult ADHD and QA, a non-parametric Spearman partial correlation was used to derive the correlation between T-scores of DSM-IV hyperactive–impulsive and DSM-IV inattentive score and QA, and the effects of FIQ, age, and sex were eliminated by using a multiple regression model. A T-score threshold of 3 was assigned and tracked using a deterministic fiber tracking algorithm (19) to obtain correlational tractography. The QA values were normalized. The seeding was placed on the whole brain. The tracks were filtered by topology-informed pruning (20) with four iteration(s). A false discovery rate (FDR) threshold of 0.05 was used to select tracks. A total of 4,000 randomized permutations were applied to the group label to obtain the null distribution of the track length to estimate the FDR.

## 3. Results

### 3.1. Dimorphic data

The Shapiro–Wilk test indicated that the data were consistent with a normal distribution at a significant level of 0.05. Levene's test indicated that the equality of the error variances was assumed at a significant level of 0.05. Table 1 shows the demographic and clinical characteristics of patients with adult ADHD and HCs. All participants were unrelated Japanese. Data obtained from 19 patients with adult ADHD (mean age: 23.89, men:women = 11:8) and 19 HCs (mean age: 25.00, men:women = 11:8) were analyzed (Table 1). Regarding the type of ADHD, 11 patients belonged to the inattentive type, and eight patients belonged to the combined type. Patients with adult ADHD and HCs did not differ significantly in age, gender, and handedness (Table 1). Patients with adult ADHD exhibited significantly lower IQ and higher CAARS than those with HCs (Table 1).

**Table 1:** Demographics of participants.

	**Health control**	**Adult ADHD**	**P-value (Two sample *t*-test, Chi-square test)**
**Demographic variables**			
Sex (Male: Female)	11: 8	11: 8	1
Agen (mean s.d)	23.89 (2.33)	25.00 (3.52)	0.262
Handedness (Right: Left)	18: 1	17: 2	1
DSM-IV inattentive symptoms (mean s.d)	48.47 (6.65)	87.05 (4.56)	*P* < 0.001
DSM-IV hyperactive/impulsive (mean s.d)	49.63 (6.66)	70.57 (16.32)	*P* < 0.001
DSM-IV total symptoms (mean s.d)	48.89 (5.87)	83.21 (7.81)	*P* < 0.001
ADHD index (mean s.d)	48.05 (5.73)	77.05 (8.23)	*P* < 0.001
Verbal IQ	122.68 (8.02)	108.63 (15.93)	0.0015
Performance IQ	114.57 (11.10)	101.2 (13.10)	0.0017
Full IQ	121.15 (7.88)	106.10 (14.84)	0.00039

### 3.2. Global network metrics based on the graph theory

#### 3.2.1. Diagnostic effects on global network metrics

Except for the clustering coefficient in the ADHD and HCs, the Shapiro–Wilk test indicated that the data were consistent with a normal distribution at a significant level of 0.05. Levene's test indicated that the equality of the error variances was assumed at a significant level of 0.05. Therefore, a comparison between adult ADHD and HCs in the clustering coefficient was done with a Mann–Whitney *U*-test, and a correlational analysis between the ADHD score and clustering coefficient was done with a Spearman's rank-order correlation. Other parameters were tested by parametric analysis (two-sample *t*-test and Pearson's correlation analysis). Table 2 shows the results of the graph theoretical analysis. We found a statistically significant difference between groups in the rich-club coefficient (*k* = 15) (*P* = 0.48, 95% CI = −0.143–−0.0001). Patients with adult ADHD had significantly lower rich-club coefficients compared with HCs. Statistically significant differences between groups in the network density, global efficiency, clustering coefficient, characteristic path length, and small worldness were not found.

**Table 2:** Results of graph theoretical analysis.

**Network parameters of graph analysis**	**Health control**	**Adult ADHD**	**P-value (95% Confidence interval)**
	**mean (standard deviation)**	**mean (standard deviation)**	
Graph density	0.388 (0.039)	0.377 (0.057)	0.473 (−0.043–0.0201)
Global efficiency	0.0938 (0.011)	0.0871 (0.015)	0.125 (0.087–0.0938)
Clustering coefficient	0.029 (0.003)	0.029 (0.007)	0.506*
Characteristic path length	14.637 (1.896)	16.186 (3.257)	0.081 (−0.205–3.3021)
Small worldness	0.0019 (0.0004)	0.0018 (0.0006)	0.529 (−0.00045–0.00023)
**Rich club**			
*K* = 5	0.999 (0.0028)	0.995 (0.0119)	0.166 (−0.0096–0.0017)
*K* = 10	0.960 (0.0333)	0.9408 (0.0725)	0.283 (−0.057–0.017)
*K* = 15	0.8202 (0.0690)	0.7484 (0.1376)	0.049 (−0.143–−0.0001)
*K* = 20	0.5849 (0.1015)	0.5385 (0.1476)	0.267 (−0.051–0.1204)

^*^Tested by Mann-Whitney *U*-test.

#### 3.2.2. Relationship between ADHD symptoms and global network metrics in patients with adult ADHD

**Figure 1:**
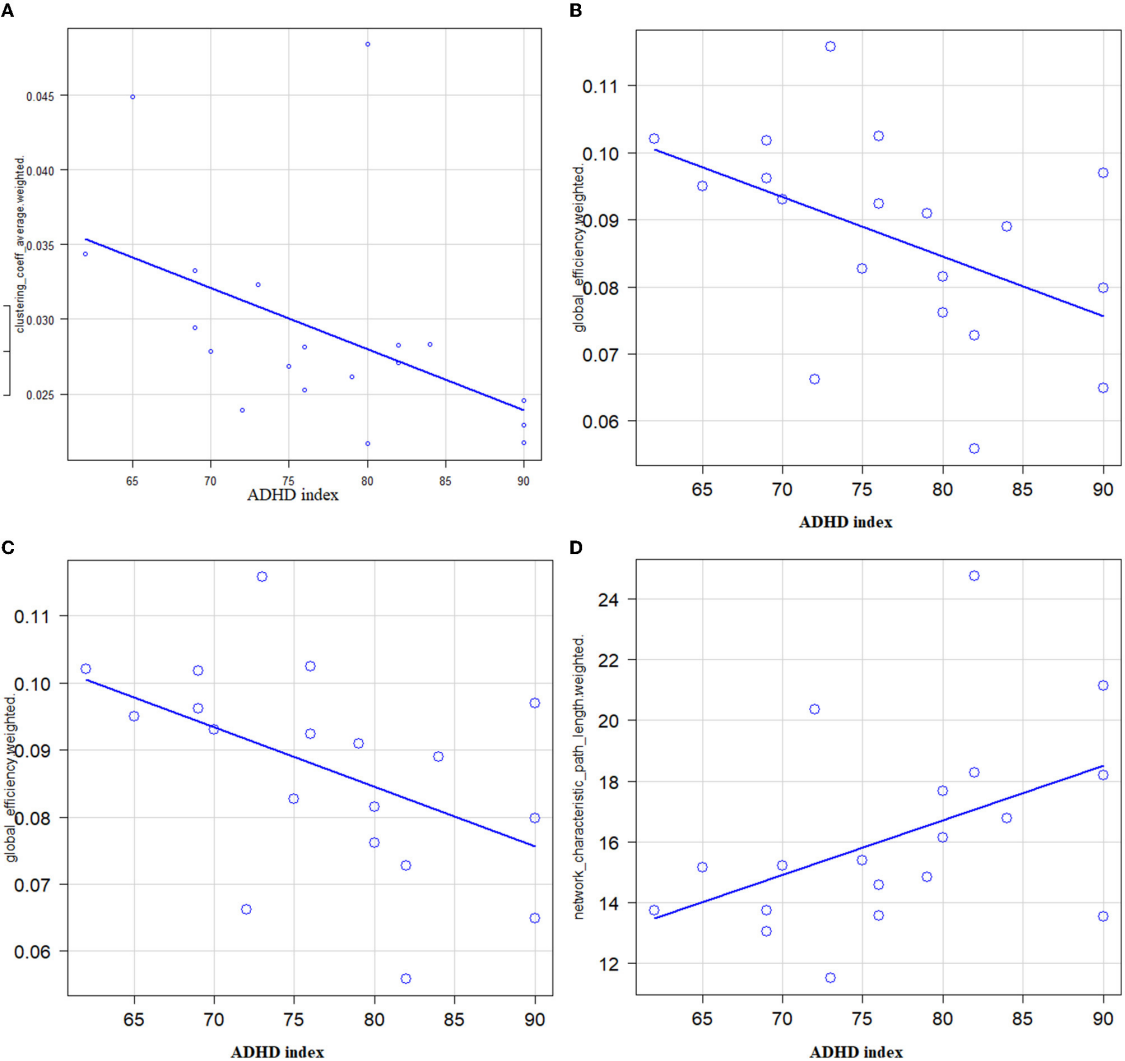
Correlations between ADHD symptoms (X: T-score of ADHD index) and global network metrics (Y) in patients with adult ADHD. **(A)** A significantly negative correlation between the T-score of the ADHD index and the global efficiency was noted (*r* = −0.481, 95% CI −0.768–−0.0346, *P* = 0.037). **(B)** A significantly negative correlation between the T-score of the ADHD index and the clustering coefficient was noted (*r* = −0.56, *P* = 0.0127). **(C)** A significantly negative correlation between the T-score of the ADHD index and the small worldness was noted (*r* = −0.603, 95% CI −0.83–−0.205, *P* = 0.0062). **(D)** A significantly positive correlation between the T-score of the ADHD index and the characteristic path length was noted (*r* = 0.454, 95% CI 0.000629–0.753, *P* = 0.0495).

There were negative correlations between the T-score of the ADHD index and global efficiency (*r* = −0.481, 95% CI −0.768–−0.0346, *P* = 0.037) (Figure 1, upper left), clustering coefficient (*r* = −0.56, *P* = 0.0127, Spearman's rank-order correlation) (Figure 1, upper right), and small worldness (*r* = −0.603, 95% CI −0.83–−0.205, *P* = 0.0062) (Figure 1, lower left). There was a positive correlation between the *T*-score of the ADHD index and characteristic path length (*r* = 0.454, 95% CI 0.000629–0.753, *P* = 0.0495) (Figure 1, lower left). There were no statistically significant correlations between the T-score of the ADHD index and the rich-club coefficient. There were no significant correlations between inattentive symptoms, hyperactive/impulsive, DSM-IV total scores, and global network metrics.

### 3.3. Local connectometry

#### 3.3.1. Diagnostic effects on connectometry

**Figure 2:**
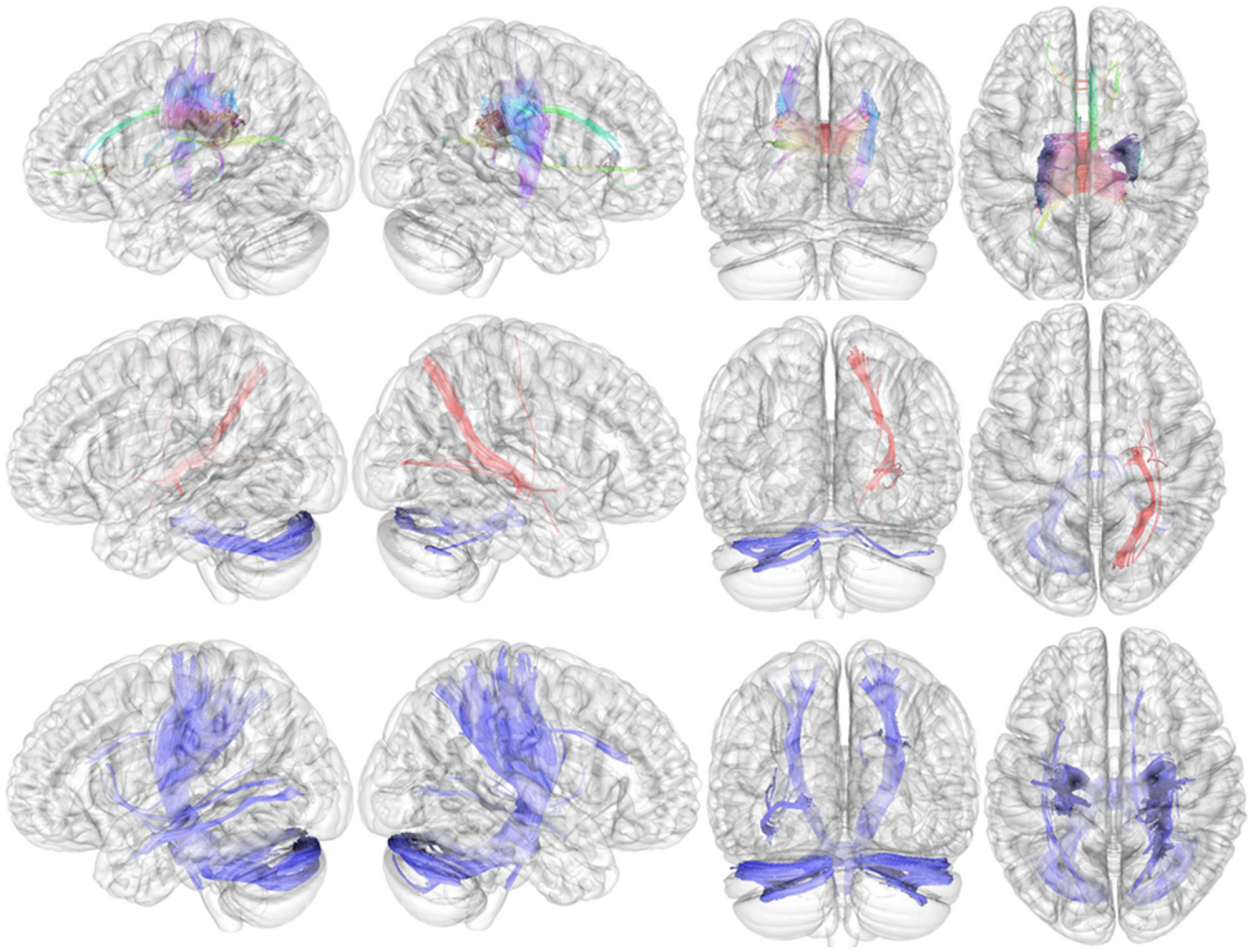
Results of local connectometry. Upper: Compared with HCs, the connectometry analysis identified significantly decreased connectivity in patients with adult ADHD in the body of the corpus callosum, the cingulum bundle, the forceps minor, the forceps major, the left fornix, the right corticospinal tract, the right superior longitudinal fasciculus III, the right medial lemniscus, and the right corticopontine tract. Middle: Significantly positive correlations between QA and severity of hyperactive–impulsive symptoms in the right corticostriatal tract, the right corticopontine tract, the right corticospinal tract, the right inferior fronto-occipital fasciculus, and the right extreme capsule (red fibers). The QA in the middle cerebellar peduncle and the left cerebellum was found to be negatively related to the severity of hyperactive–impulsive symptoms (blue fibers). Bottom: The connectometry analysis found negative correlations between QA and severity of inattentive symptoms in the bilateral corticospinal tract, the left inferior longitudinal fasciculus, the right corticopontine tract, the middle cerebellar peduncle, the bilateral cerebellum, the right medial lemniscus, and the right dentatorubrothalamic tract.

Compared with HCs, the connectometry analysis identified significantly decreased connectivity in patients with adult ADHD in the body of corpus callosum, the cingulum bundle, the forceps minor, the forceps major, the left fornix, the right corticospinal tract, the right superior longitudinal fasciculus III, the right medial lemniscus, and the right corticopontine tract (Figure 2, upper). The connectometry analysis identified no track with increased connectivity in patients with adult ADHD.

#### 3.3.2. Connectivity associated with symptoms of ADHD

In patients with adult ADHD, positive correlations between QA and severity of hyperactive/impulsive symptoms (*T*-score of DSM-IV hyperactive–impulsive symptoms) were observed in the right corticostriatal tract, the right corticopontine tract, the right corticospinal tract, the right inferior fronto-occipital fasciculus, and the right extreme capsule (Figure 2, middle; red fibers). The QA in the middle cerebellar peduncle and left cerebellum was found to be negatively related to the severity of hyperactive–impulsive symptoms (Figure 2, middle; blue fibers).

The connectometry analysis found negative correlations between QA and severity of inattentive symptoms in the bilateral corticospinal tract, the left inferior longitudinal fasciculus, the right corticopontine tract, the middle cerebellar peduncle, the bilateral cerebellum, the right medial lemniscus, and the right dentatorubrothalamic tract (Figure 2, bottom; blue fibers). There was no significant result in tracks with QA positively correlated with the severity of inattentive symptoms.

On the contrary, there was no significant result in tracks with the QA correlated with the ADHD index.

## 4. Discussion

In this study, we performed comprehensive analyses of DTI data, local connectometry analysis by using QA and graph theoretical analysis, and conducted comparisons of several measurements between patients with HCs and patients with adult ADHD. We first discuss the results of global network metrics estimated by graph theory and then discuss the results of local connectometry.

### 4.1. Global network metrics

Patients with adult ADHD had a significantly lower rich-club coefficient than HCs. Other indicators, such as small worldness and global efficiency, which were found to be abnormal in previous studies of children and adolescents with ADHD (21, 22), were not significantly different in the present study. Network hubs that are members of the rich club are connected to each other, establishing a central rich club that serves as a hub for interregional and global neural signaling as well as whole-brain integration and communication (18). The results of the graph theoretical analysis suggest that adults with ADHD have altered functional integration and global information communication in the brain. While children with ADHD have a deteriorated small-world network structure, with lower global efficiency and higher local efficiency (21–23), there were discrepancies between studies in the results of global network measures in patients with adult ADHD (23–27). Sidlauskaite et al. reported no differences between patients with adult ADHD and HCs in terms of global network metrics, such as small worldness, global efficiency, and clustering coefficient (24). Meanwhile, other studies reported lower global efficiency, abnormal rich-club organization and reduced hemispheric asymmetry in adult ADHD (25, 27). However, these three studies of positive results of global network metrics were reported from the same country, and two out of three studies were from the same laboratory. The results of this study, abnormal rich-club organization, and possible lower global communication in patients with adult ADHD, are similar to the results of those studies and may support the positive results of previous studies (25, 27). Correlational analyses revealed correlations between global network metrics and the overall severity of symptoms. The more severe the overall symptoms of ADHD, global efficiency, clustering coefficient, and small worldness were lower, and characteristic path length was longer, suggesting that altered functional integration and global information communication in patients with adult ADHD contribute to the general symptomatic severity of ADHD and its close relationship with the pathophysiology of ADHD. Although patients with adult ADHD showed a lower rich-club coefficient than HCs, the correlational analysis showed no correlation between the rich-club coefficient and symptomatic severity. This appears to be some sort of contradictory result. We cannot provide a clear explanation at this time, but we speculated that the rich-club coefficient abnormality may be a trait marker rather than a state marker of ADHD. Further studies will be needed to clarify such speculation.

### 4.2. Local connectometry

The local connectometry revelated decreased connectivity in the corpus callosum (CC) (body) and fibers near the corpus callosum, such as the forceps minor, the forceps major, the cingulum bundle, and the left fornix in patients with adult ADHD. In addition, the right corticospinal tract, the right corticopontine tract, the right medial lemniscus, and the right superior longitudinal fasciculus III (SLF III) also showed decreased connectivity in patients with adult ADHD. We speculated that widely distributed disrupted structural connectivity including the CC revealed by local connectometry could be related to the less efficient information transfer system revealed by graph theory in patients with adult ADHD and related to overall symptoms of ADHD. Except for median structure such as the CC and the cingulum, the white matter fiber abnormalities in adult ADHD in this study were found in the right hemisphere as in previous studies (23), suggesting a contribution to the problem of hemispheric lateralization problems in ADHD (25, 27).

The CC is responsible for hemispheric lateralization and communication between cerebral hemispheres (28). Studies of connectome with agenesis of the corpus callosum demonstrated that patients with agenesis of the corpus callosum showed longer path length and lower global efficiency (29, 30). This suggests that CC abnormalities may contribute to global network metrics that reflect functional integration. The abnormalities of CC in patients with ADHD have been consistently reported by previous studies, including meta-analyses (23). The forceps major connects the bilateral occipital lobes *via* the splenium of the corpus callosum, and the forceps minor connects the lateral and medial surfaces of the frontal lobes *via* the genu of the corpus callosum (28). Several studies, including in patients with adult ADHD, showed abnormality in the forceps major and minor (23, 31). A previous study demonstrated the relationship between executive function and FA in the the right forceps minor in bilingual young adults and showed higher FA in the forceps minor predicted the high performance of a task requiring executive function (32). Moreover, a previous study reported that the disintegrity of the white matter in both forceps major and minor contributed to more declined cognitive function in patients with type-2 diabetes (33). These data suggest that impairment in the forceps contributes to cognitive disability, including executive function, which is often observed in patients with ADHD.

The cingulum bundle and fornix are important parts of the limbic system (28, 30). According to DTI studies, the cingulum takes longer to mature even after adolescence and frequently does not take on adult traits until the mid-20s or later (34, 35). Neuroimaging studies have demonstrated that functions of the cingulum bundle are related to executive/attention functions, emotion, and memory, while clinical studies have demonstrated cingulum abnormalities in neuropsychiatric diseases, including ADHD (36). The cingulum bundle and fornix are important components of the Papez circuit, which is believed to be essential for cognition, emotion, and episodic memory (36, 37). The cingulum bundle connects the anterior thalamic nucleus to the cingulate gyrus, and it also connects the cingulate gyrus to the parahippocampal region, while the fornix connects the subiculum to the mammillary bodies (36, 37). Therefore, the Papez circuit may also play a role in the pathophysiology of major depressive disorders (36, 38). Although there were no obvious psychiatric comorbidities in our sample, adult ADHD is often associated with psychiatric comorbidities and a high prevalence of depression has also been reported (39). Common genetic factors for ADHD and depression have been reported as contributing to this phenomenon (40). Therefore, we speculate that abnormalities of these regions seem to be associated with not only cognitive disability but also susceptibility to depressive disorder in adult ADHD. Indeed, in depression, abnormalities in the cingulum bundle and fornix have been observed (36, 38).

The corticopontine tract and the corticospinal tract are associated with the coordination of planned motor functions (23, 28), and abnormalities of these tracts have been considered to be associated with deficits in fine motor control in patients with ADHD (23). However, a recent study found that the corticopontine tract, a component of cortical-ponto-cerebellar pathways, also known as the cerebello-thalamo-cortical or cortico-ponto-cerebellar loop, serves as a medium for cerebro-cerebellar communication during cognitive processing (41). We speculated that abnormality in the corticopontine tract might be associated with not only motor control disorder but also cognitive disability in patients with ADHD. The SLF III had possible connections between the supramarginal gyrus and the pars opercularis and bidirectional connections between the ventral prefrontal cortex and the inferior parietal lobule (28). The SLF III may function to transfer somatosensory information, including language articulation *via* monitoring orofacial and hand motions (28). The abnormalities in the SLF were also reported in patients with ADHD (23).

This study found disintegrity in the right medial lemniscus in adult ADHD. To the best of our knowledge, there has been no report of abnormality in this region in patients with ADHD; however, a previous study reported decreased FA in this region in patients with autistic spectrum disorder (ASD) (42). The medial lemniscus serves as a major route for ascending sensory fibers to the ventroposterolateral thalamus (28). Since ADHD and ASD are often concurrent and shared sensory symptoms such as hyper- and hyposensitivity to various types of sensory input (43), abnormality in the medial lemniscus might be associated with sensory symptoms in ADHD.

Although global network metrics did not significantly correlate with hyperactive/impulsive or inattentive symptoms in the current study, local connectometry detected abnormal fibers associated with these symptoms. Regarding the hyperactive–impulsive symptoms, severe symptoms are associated with higher QA (suggesting overconnectivity) in the right corticostriatal tract, the corticospinal tract, vextreme capsule, and the right inferior fronto-occipital fasciculus. The corticostriatal projections are critical components of forebrain circuits that are extensively involved in motivated behavior (28). Not only decreased connectivity but also overconnectivity may also disrupt structural connectivity and information transfer, and some studies demonstrated overconnectivity in patients with ASD (40). The corticostriatal circuits have been linked to dopaminergic pathways that connect the striatum to the prefrontal cortex and other areas, and dysfunction of these circuits has been linked to impairments in cognitive functions and the ability to adapt behavior to changing circumstances (44). Considering the function of the corticostriatal tract and its contribution to the pathophysiology of ADHD, the association between abnormality in this region and hyperactive–impulsive symptoms seems to be plausible. The inferior fronto-occipital fascia (IFOF) is a WM tract that originates in the occipital and parietal lobes, ends in the inferior frontal lobe, and is connected to the inferolateral insula *via* the extreme and external capsules with the uncinate fasciculus. While the IFOF and extreme capsule are primarily associated with semantic language processing and transmission (28), research has shown that the IFOF connects the salience network to the executive control network, a possible serving role in goal-oriented behavior (45). We speculated that, together with the corticostriatal tract abnormalities, IFOF abnormalities may contribute to behavioral abnormalities, such as impulsiveness in ADHD-based disability of goal-oriented behaviors.

Decreased connectivity in the cerebellum was associated with both hyperactive–impulsive and inattentive symptoms. As mentioned previously, cerebro-cerebellar communication should be an important component in cognitive processing (38). Although dopamine receptors are not abundant in the cerebellum, it is involved in the indirect regulation of dopaminergic neurotransmission (44). An animal study demonstrated that the vermis and paravermal areas in the cerebellum modulate the rate of dopamine and noradrenaline turnover in the caudate and nucleus accumbens (44). Considering the intimate relationship between the dopaminergic system and the pathophysiology of ADHD, the association between abnormality in the cerebellar circuit and symptoms of ADHD seems to be plausible. Indeed, many studies have demonstrated abnormalities of the cerebellum in ADHD (23, 46).

The results for corticospinal and corticopontine tracts were ambiguous. Compared with HCs, these tracts showed significantly decreased connectivity in the adult ADHD group, but correlational analysis showed a positive correlation with the severity of hyperactive/impulsive symptoms. Furthermore, the corticospinal and corticopontine tracts showed opposite QA patterns in association with symptoms. As described earlier, higher QA in these areas was related to the severity of hyperactive–impulsive symptoms, whereas lower QA was related to the severity of inattentive symptoms. We have no clear explanation of these phenomena but speculate that different patterns of white matter abnormalities, decreased connectivity, and overconnectivity in the same region might be associated in different ways and might contribute to different symptoms in ADHD and patterns of the combination of overconnectivity/decreased connectivity of white matter fibers in each region, which might be associated with severity of clinical symptoms. However, there are no previous studies to support these speculations at this time, and further research is needed. Regarding other tracts, the inferior longitudinal fasciculus (associated with object recognition, face recognition, lexical and semantic processes, emotion, and visual memory) (47), the medial lemniscus (associated with sensory processing) (28), and the dentatorubrothalamic tract (associated with motor control and also is a known site of deep stimulation for the treatment of tremor) (28, 48), we have no clear explanation for associations between disintegrity in these fibers and severity of inattentive symptoms at present because there has been no study that reported a direct relationship between attention and these regions.

### 4.3. Strengths and limitations of this study

The superior point of this study is the strict selection of treatment-naive patients with adult ADHD. Since WM integrity was affected by the treatment of MPH, it was important to evaluate DTI data in treatment-naive patients with adult ADHD.

Meanwhile, there are some limitations in this study. First, as already mentioned in the Discussion section, the results for the corticospinal and corticopontine tracts were ambiguous, and we have no clear explanation for the phenomenon. Further research is needed to clarify such phenomena. The second limitation is a relatively small sample size of the study. Although Friston's loss-function analysis suggests that the optimal sample size for a neuroimaging study is 16 to 32 participants (49, 50), the small sample size in this study may underestimate the detection of changes in connectivity due to a lack of statistical power and may contribute to seemingly contradictory results, such as the difference between correlation analysis and group comparison results, and due to the fact that graph theory correlation analysis showed a correlation only with the ADHD index but no correlation with core symptoms. However, our findings are similar to previous studies with a larger sample size. Third, this study is a biological study with a small sample, and therefore, we excluded adult ADHD patients with comorbid psychiatric disorders. On the contrary, patients with adult ADHD have been reported to have a high frequency of various comorbid disorders, including affective disorders, anxiety disorders, and substance abuse (39, 51). Therefore, there are limitations in generalizing the results of this study to patients with adult ADHD, and this point should be interpreted with caution. Fourth, the maximum b-value and the number of directions in this study were lower than those recommended by DSI Studio (13). We cannot deny the possibility of reduced sensitivity in detecting WM due to our DTI acquisition parameters. However, previous studies that performed DTI like the DTI acquisition parameters in our study and performed similar analyses using DSI Studio demonstrated pathologically relevant results (52, 53). Finally, healthy control participants in the present study had high IQs. Due to the existence of the relationship between white matter development and connectome and intelligence (54, 55), control participants with high IQ affected the results of this study. However, correlational analyses were conducted only on participants with adult ADHD, and we found relevant results in terms of the pathophysiology of ADHD.

## 5. Conclusion

Treatment-naive patients with adult ADHD showed lower rich-club coefficients and decreased connectivity of the corpus callosum and fibers near the corpus callosum and several fibers in the right cerebral hemisphere. The widely distributed abnormal WM would contribute to the abnormal rich club and consequently less efficient information transfer in the ADHD brain. The severity of overall ADHD symptoms was associated with several global network metrics and several fibers associated with the severity of the hyperactive–impulsive symptom and the inattentive symptom, suggesting the contribution of WM abnormalities in the adult ADHD brain to the pathophysiology of adult ADHD.

## Data availability statement

The datasets presented in this article are not readily available because Due to the nature of this research, participants of this study did not agree for their data to be shared publicly, so supporting data is not available. Requests to access the datasets should be directed to tohnish8@its.jnj.com.

## Ethics statement

The studies involving human participants were reviewed and approved by Research Ethical Committee of Fukushima Medical University. The patients/participants provided their written informed consent to participate in this study.

## Author contributions

TO: conceptualization, methodology, data analyses, writing—original draft preparation, and project administration. HY: writing—review and editing and supervision. HI, SIs, AS, and IM: data collection and writing—review and editing. JM: writing—review and editing. WT and SIt: conceptualization, data collection, and writing—review and editing.
